# Early Morning Blood Draw Timing and Frequency Among Hospitalized Patients

**DOI:** 10.1001/jamanetworkopen.2026.0966

**Published:** 2026-03-11

**Authors:** Michael Colacci, Anne Loffler, Surain B. Roberts, William K. Silverstein, Adina Weinerman, Amol A. Verma, Fahad Razak

**Affiliations:** 1 Li Ka Shing Knowledge Institute, St Michael’s Hospital, Toronto, Ontario, Canada; 2 Department of Medicine, University of Toronto, Toronto, Ontario, Canada; 3 Institute of Health Policy, Management, and Evaluation, University of Toronto, Toronto, Ontario, Canada; 4 Division of General Internal Medicine, Sunnybrook Health Sciences Centre, Toronto, Ontario, Canada

## Abstract

This cohort study evaluates the frequency of early morning blood draws in 18 hospitals in Ontario, Canada, and assesses variability in phlebotomy practices between institutions and over time.

## Introduction

Hospitalized patients report less sleep in the hospital and an increased number of nocturnal awakenings, which has been associated with the development of delirium.^1,2^ Among the most prevalent and easily modifiable causes of nocturnal awakenings are early morning blood draws.^1^ A prior study at a single institution identified that nearly 40% of blood draws occur between 4:00 and 7:00 am and may directly contribute to sleep disruption.^3^ It remains unclear whether early morning phlebotomy is common practice in other health care jurisdictions and across a large sample of hospitals. The objective of this study was to evaluate the frequency of early morning blood draws in 18 hospitals in Ontario, Canada, and to assess variability in phlebotomy practices between institutions and over time.

## Methods

We conducted a retrospective cohort study of adults hospitalized on general medicine units at 18 Ontario hospitals participating in the GEMINI research network between April 1, 2015, and June 30, 2022, following the STROBE reporting guidelines.^4,5,6^ This study was approved by the research ethics board of all participating hospitals, which waived the requirement for individual patient consent.

We included all inpatient blood draws, except those completed in the emergency department, during the first 24 hours of admission, and in the intensive care unit, as these are typically completed on an urgent basis.^3^ Early morning blood draws were defined as those occurring between 4:00 and 7:00 am.^3^

Outcomes included the proportion of total blood draws that occurred between 4:00 and 7:00 am and the proportion of encounters with at least 1 early morning blood draw. We examined the frequency of early-morning blood tests over time across the full cohort and by hospital. Temporal trends were assessed using mixed-effects negative binomial regression with discharge quarter as a fixed effect and hospital-level random effects. We conducted a sensitivity analysis restricted to routinely collected blood tests (sodium and hemoglobin). Data were analyzed with R statistical software version 4.2.2 (R Project for Statistical Computing). Additional details are shown in the eAppendix in Supplement 1.

## Results

Among 526 881 hospitalizations across 18 hospitals with 3 582 231 unique blood draws (134 929 898 total blood tests), the median (IQR) patient age was 73 (59-84) years, 262 760 patients (49.9%) were female, the median (IQR) length of stay was 6.2 (3.5-11.9) days, and the most common admission diagnosis was heart failure (31 012 patients [5.9%]). The median (IQR) number of blood draws per hospitalization was 4 (2-8), and the number per day was 0.8 (0.5-1.0).

Overall, 1 078 134 blood draws (30.3%) occurred between 4:00 and 7:00 am, and 298 820 admissions (56.7%) included at least 1 early morning blood draw (median [IQR] per hospitalization, 1 [0-2] blood draws). The proportion of early morning blood draws decreased slightly over time, from 31.0% (95% CI, 30.9%-31.2%) in 2015, to 29.4% (95% CI, 29.3%-29.5%) in 2022 (Figure), with no significant change during the first 2 years of the COVID-19 pandemic. Hospitalizations with early morning blood draws had slightly higher median (IQR) modified Laboratory-based Acute Physiology Scores at admission (20 [9-33] vs 17 [6-29]; standardized mean difference, 0.184) (Table).

**Figure.**
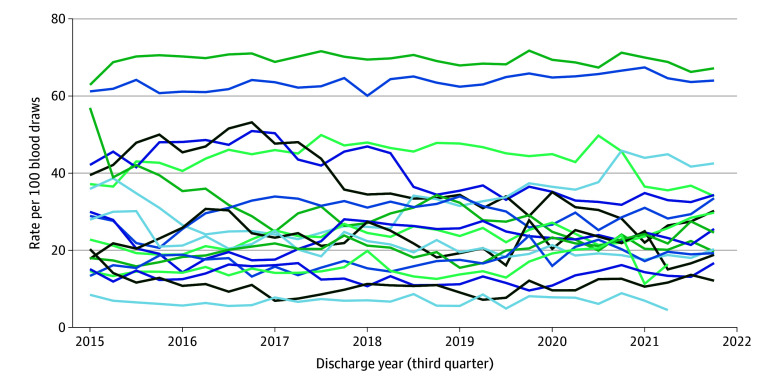
Graph of Frequency of Early Morning Blood Draws Across Hospitals, 2015 to 2022 The y-axis is the rate of blood draws that occurred between 4:00 and 7:00 am, per 100 blood draws. The x-axis is the calendar date. Each line corresponds to an individual hospital.

**Table.** Baseline Characteristics of the Entire Cohort and Stratified by Receipt of an Early Morning Blood Test

Characteristic	Patients, No. (%)			SMD^a^
	Total cohort (N = 526 881)	Encounters with no early morning blood draw (n = 228 061)	Encounters with an early morning blood draw (n = 298 820)	
Age, median (IQR), y	73.0 (59.0-84.0)	73.0 (58.0-84.0)	73.0 (59.0-84.0)	0.032
Sex				
Female	262 760 (49.9)	114 251 (50.1)	148 509 (49.7)	0.008
Male	264 121 (50.1)	113 810 (49.9)	150 311 (50.3)	
Modified Laboratory-based Acute Physiology Score, median (IQR)	18.0 (7.0-31.0)	17.0 (6.0-29.0)	20.0 (9.0-33.0)	0.184
Charlson Comorbidity Index score				
0	247 417 (47.0)	113 029 (49.6)	134 388 (45.0)	0.095
1	99 848 (19.0)	42 163 (18.5)	57 685 (19.3)	
≥2	179 616 (34.1)	72 869 (32.0)	106 747 (35.7)	
Resides in long-term care facility	47 606 (9.0)	21 018 (9.2)	26 588 (8.9)	0.011
Admitted from emergency department	494 140 (93.8)	214 278 (94.0)	279 862 (93.7)	0.012
Admitted on weekend	140 821 (26.7)	59 920 (26.3)	80 901 (27.1)	0.018
Admitted at night (5:00 pm to 8:00 am)	380 551 (72.2)	161 438 (70.8)	219 113 (73.3)	0.057
No. of blood draws/d (after first 24 h), median (IQR)	0.8 (0.5-1.0)	0.8 (0.5-1.0)	0.9 (0.5-1.0)	0.250

Abbreviation: SMD, standardized mean difference.

^a^ An SMD less than 0.1 indicates balance between groups. There were no missing data.

Across hospitals, the proportion of early morning blood draws ranged from 6.9% (10 687 of 154 191 patients) to 69.4% (193 082 of 278 299 patients), and the proportion of admissions with at least 1 early morning blood draw ranged from 16.1% (3608 of 22 443 patients) to 95.8% (32 615 of 34 051 patients). Eight hospitals decreased (range, 0.4%-13.2% decrease per year) and 8 increased (range, 1.2%-7.2% increase per year) early morning blood draw utilization. Findings were similar in a sensitivity analysis restricted to sodium and hemoglobin measurements.

## Discussion

In this multicenter cohort study of over 3.5 million blood draws from 526 000 hospitalizations across 18 Ontario hospitals, early morning blood draws were common, affecting more than one-half of general medicine admissions. Practice varied widely across hospitals (6.9% to 69.4%) and over time, with nearly one-half of hospitals demonstrating increases or decreases in early morning testing, suggesting opportunities for standardization. Observed variation likely reflects differences in phlebotomy staffing and workflows, physician practices, and quality improvement initiatives. Limitations include the retrospective design, uncertainty regarding factors underlying variation, and lack of linkage to patient outcomes.

Despite growing awareness of their impact on sleep and patient experience, early morning blood draws remain prevalent. Reducing their frequency, particularly among high-risk groups such as patients with delirium, represents an opportunity to improve patient-centered care.

## Notes

Supplement 1. eAppendix. Supplemental Methods eReferences

Supplement 2. Data Sharing Statement
