# Supplementary material for: Early Morning Blood Draw Timing and Frequency Among Hospitalized Patients
Source: JAMA Netw Open. 2026 Mar 11;9(3):e260966. doi: 10.1001/jamanetworkopen.2026.0966 (PMC12980245; doi:10.1001/jamanetworkopen.2026.0966)
Supplement: Supplement 1. — eAppendix. Supplemental Methods eReferences [file jamanetwopen-e260966-s001.pdf]

Supplemental Online Content

Colacci M, Loffler A, Roberts SB, et al. Early morning blood draw timing and frequency among hospitalized patients. *JAMA Netw Open*. 2026;9(3):e260966. doi:10.1001/jamanetworkopen.2026.0966

eAppendix. Supplemental Methods
eReferences

This supplemental material has been provided by the authors to give readers additional information about their work.

© 2026 Colacci M et al. *JAMA Network Open*.

eAppendix. Supplemental Methods

Study Population

Ontario is the largest single-payer health system in North America with a population of >16 million people, and hospitals in the GEMINI network encompass over 50% of medical hospitalizations in Ontario.[1] The included hospitals encompass a diverse and representative hospital sample including academic medical centers, community hospitals, urban and rural facilities, and institutions of varying sizes across all six provincial health regions. We have previously completed a large-scale descriptive study of the included population published in JAMA Network Open (PMID: 39813029) which provides additional background information.

Measures and Definitions

Administrative and clinical data were extracted with high accuracy from each patient's electronic health records through previously described methods.[2] We measured baseline clinical characteristics and hospitalization outcomes for each general medicine admission, including the modified Laboratory Acute Physiology Score (mLAPS), a measure of illness acuity.[3] The medical record captures the number of unique blood tests but not the number of blood draws. The timing of a blood draw was assigned using the earliest laboratory test collection time for that blood draw. To estimate blood draws, we assumed that all laboratory tests performed within a 30-minute window were collected from a single draw/venipuncture and were therefore counted as a single blood draw. This assumption reflects standard clinical practice, where unique blood draws are rarely performed more frequently than every four hours on a general medicine ward.

© 2026 Colacci M et al. *JAMA Network Open*.

The primary outcomes included the proportion of total blood draws that occurred between 4:00-7:00am and the proportion of encounters with at least one early morning blood draw. Secondary outcomes included the median number of days with an early morning blood draw per encounter, as well as the variability in proportion of early morning blood draws between individual hospitals and over time.

Statistical Analysis

We present descriptive statistics for baseline clinical characteristics and outcomes grouped into encounters that did and did not have an early morning blood draw.[4]

Mixed effects negative binomial regression model: The outcome was the number of early-morning blood draws per encounter, with the total number of blood draws included as an offset using its natural logarithm.

© 2026 Colacci M et al. *JAMA Network Open*.

eReferences

1. Statistics Canada. 2023. (table). Census Profile. 2021 Census of Population. Statistics Canada Catalogue no. 98-316-X2021001. Ottawa. Released March 29, 2023. https://www12.statcan.gc.ca/census-recensement/2021/dp-pd/prof/index.cfm?Lang=E (accessed April 25, 2023).
2. Verma AA, Pasricha S V., Jung HY, et al. Assessing the quality of clinical and administrative data extracted from hospitals: The General Medicine Inpatient Initiative (GEMINI) experience. *Journal of the American Medical Informatics Association*. 2021;28(3). doi:10.1093/jamia/ocaa225
3. Roberts SB, Colacci M, Razak F, Verma AA. An Update to the Kaiser Permanente Inpatient Risk Adjustment Methodology Accurately Predicts In-Hospital Mortality: a Retrospective Cohort Study. *J Gen Intern Med*. Published online June 9, 2023. doi:10.1007/s11606-023-08245-w
4. Austin PC. Balance diagnostics for comparing the distribution of baseline covariates between treatment groups in propensity-score matched samples. *Stat Med*. 2009;28(25):3083-3107. doi:10.1002/sim.3697

© 2026 Colacci M et al. *JAMA Network Open*.
