# Supplementary material for: Early Morning Blood Draw Timing and Frequency Among Hospitalized Patients
Source: JAMA Netw Open. 2026 Mar 11;9(3):e260966. doi: 10.1001/jamanetworkopen.2026.0966 (PMC12980245; doi:10.1001/jamanetworkopen.2026.0966)
Supplement: Supplement 2. — Data Sharing Statement [file jamanetwopen-e260966-s002.pdf]

Data Sharing Statement

Colacci. Early Morning Blood Draw Timing and Frequency Among Hospitalized Patients at 18 Hospitals. *JAMA Netw Open*. Published March 11, 2026.
doi:10.1001/jamanetworkopen.2026.0966

Data
Data available: No

Additional Information
Explanation for why data not available: The data utilized for this study is part of the GEMINI database and protected by privacy agreements. Requests for access to this data can be made at geminimedicine.ca
